# A case of autoimmune pulmonary alveolar proteinosis responding to oral statin therapy

**DOI:** 10.1016/j.rmcr.2024.102042

**Published:** 2024-05-25

**Authors:** So Shimamura, Honami Morikawa, Ken Shinohara, Hiroki Ohkoshi, Chisa Omori, Yuuki Hoshino, Yoshinori Uchida, Saiki Masafumi, Shinnosuke Ikemura, Naoki Ohishi, Tetsuo Kondo, Kenzo Soejima

**Affiliations:** a Department of Respiratory Medicine, Graduate School of Medicine University of Yamanashi, 1110 Shimokato, Chuo, Yamanashi, 409-3892, Japan; b Department of Pathology, Graduate School of Medicine University of Yamanashi, 1110 Shimokato, Chuo, Yamanashi, 409-3892, Japan

## Abstract

There is no approved drug treatment for autoimmune pulmonary alveolar proteinosis (APAP), although traditionally requires complex treatments such as whole lung lavage (WLL). We herein report on a 67-year-old man diagnosed with APAP. Treatment with atorvastatin (5 mg daily) resulted in significant improvement in symptoms, lung function, and computed tomography findings, with enhanced oxygenation, although serum anti-GM-CSF antibody levels remained elevated. This case suggests that the remission observed in this case could potentially be attributed to a direct effect of atorvastatin within the pulmonary alveoli. Statins may be considered as one of the treatment options for APAP.

## 1 Introduction

Pulmonary alveolar proteinosis (PAP) is a rare disease that results in the accumulation of excess surfactant in the alveoli and respiratory bronchioles due to a defect in the production or degradation of surfactant, leading to respiratory failure [1]. Depending on the etiology, it is classified as autoimmune pulmonary alveolar proteinosis (APAP), secondary pulmonary alveolar proteinosis (SPAP), or congenital alveolar proteinosis. The treatment of APAP is classified according to the severity of the disease and includes symptomatic treatment with expectorants and antitussives, whole lung lavage (WLL), segmental lung lavage (SLL), and the use of cough suppressants. There is no approved drug treatment for APAP, although experimental treatments such as WLL or SLL and rh GM-CSF inhalation therapy are used depending on the severity of the disease.

## 2 Case presentation

**Fig. 1:**
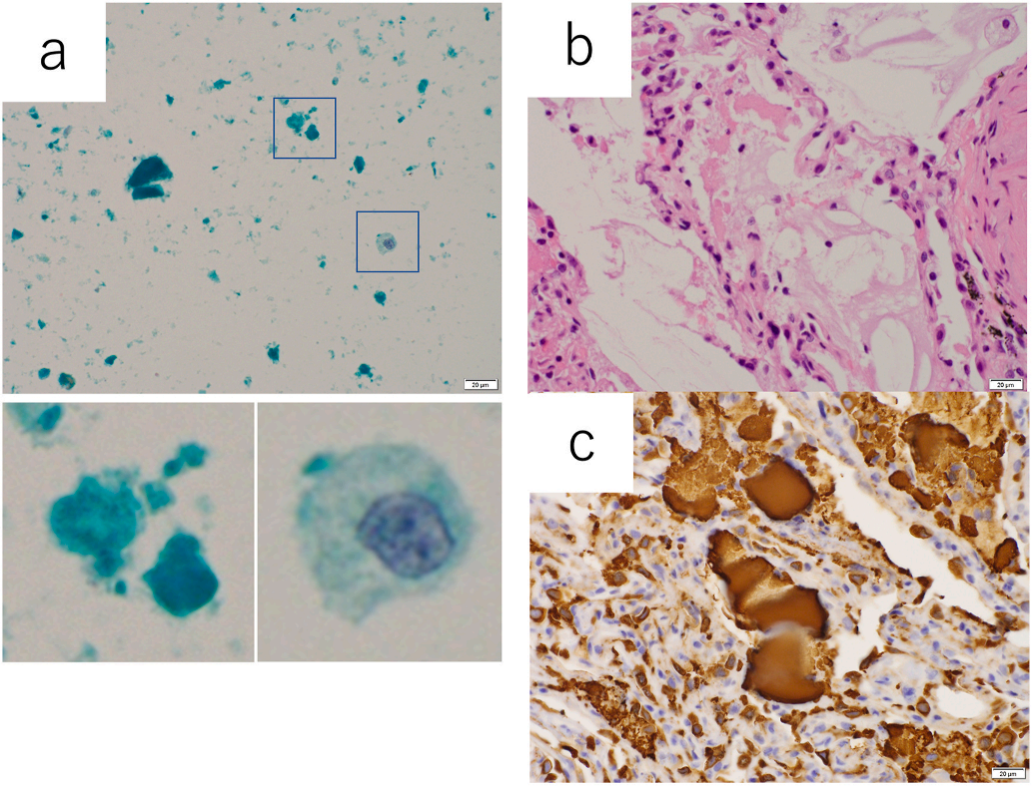
Cytology of BALF and biopsy tissue at bronchoscopy at diagnosis. (a) Papanicolaou staining revealed pale green-stained granular non-structural material and foamy macrophages. (b) Acidophilic fine granular material was found in the peripheral airspace. (c) Fine granular material was positive for SP-A by immunostaining. (For interpretation of the references to colour in this figure legend, the reader is referred to the Web version of this article.)

The patient is a 67-year-old man, who was found to have an abnormal chest X-ray during a medical check-up in August 2012. He was initially followed up at another hospital for interstitial pneumonia, but his dyspnea symptoms slowly worsened over time. His dyspneic symptoms continued to worsen, and worsening was also observed on imaging studies. The patient had a history of hypertension, and mild dyslipidemia was noted. At the time of his first visit to our hospital, he complained of dyspnea, and his oxygen saturation in the room was 93 %. On auscultation of the chest, fine crackles were heard on the bilateral dorsal thoracic surfaces. Laboratory tests showed lactate dehydrogenase level of 359 U/L, KL-6 level of 11,705 U/mL, total cholesterol level of 219 mg/dL and low-density lipoprotein cholesterol (LDL-C) level of 152 mg/dL. Chest X-ray showed bilaterally symmetrical infiltrative shadows distributed in the middle and lower lung fields, while computed tomography revealed a wide Crazy-paving pattern in the bilateral lung fields. (In addition, fibrosis of the lungs and traction bronchiectasis were observed in the bilateral lower lobes.) For a definitive diagnosis, bronchoalveolar lavage was performed from the middle lobe of the right lung and transbronchial lung biopsy from S3 and S8 of the right lung. The appearance of the BALF was milky white, and cytology showed granular non-structural material and foamy macrophages with light green staining by Papanicolaou staining. Histological examination showed acidophilic fine granular material in the peripheral airspace, which was positive for SP-A by immunostaining (Fig. 1). Serum anti-GM-CSF antibodies were abnormal (93.7 U/ml, reference value < 1.7 U/ml), leading to a diagnosis of autoimmune PAP.

**Fig. 2:**
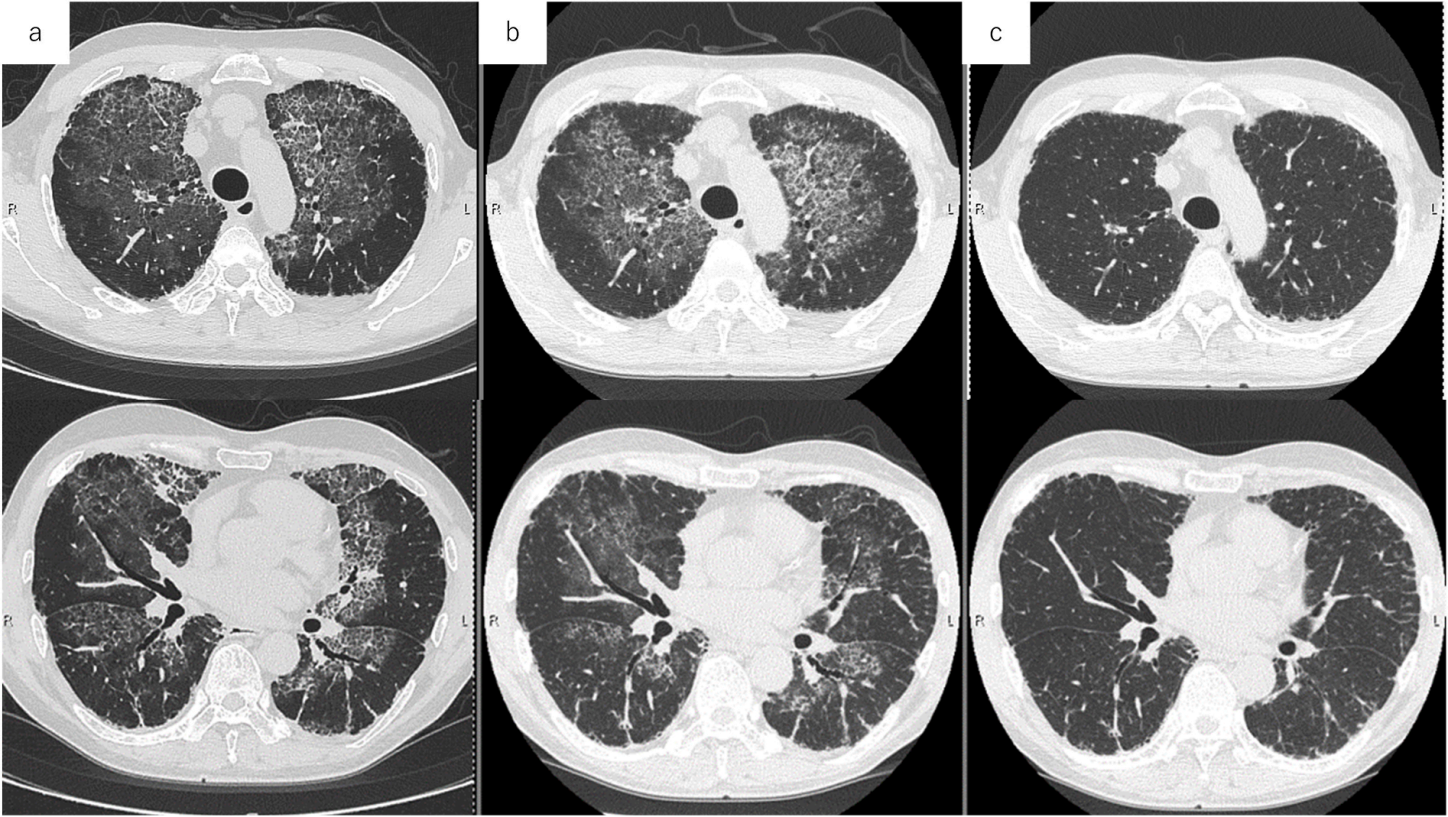
Chest CT findings in the upper and lower lung fields before and after atorvastatin administration. (a) Prior to the initiation of oral statin therapy, a diffuse crazy-paving pattern was evident in both lung fields. (b) After 8 months of oral statin treatment, improvements were observed in the ground glass and reticular changes in the bilateral lower lobes. (c) After 12 months of oral statin treatment, significant enhancements were noted in the ground glass and reticular changes throughout the entire bilateral lung field.

A blood gas test at diagnosis showed PaO_2_ of 62 Torr and AaDO_2_ of 42.2 Torr, resulting in a disease severity score (DSS) of 3 for PAP. The patient exhibited mild hypercholesterolemia, with an LDL-C level of 152 mg/dL. Upon explaining to the patient that statins, medications used for high cholesterol, might be potentially effective in treating PAP, the patient consented to initiate atorvastatin 5 mg daily in March 2022. After the initiation of statin treatment, the patient's dyspnea symptoms gradually ameliorated. Subsequent computed tomography revealed a significant improvement in the Crazy-paving pattern observed in the bilateral lung fields, which had nearly disappeared during the examination conducted in March 2023, one year after the commencement of statin treatment (Fig. 2). Blood gas test indicated a PaO_2_ of 72.1 Torr, AaDO_2_ of 27 Torr, and an improvement in AaDO_2_ by more than 10 Torr, leading to a reduction in DSS to 1. Hematological analyses demonstrated a substantial decrease in KL-6 levels. The total cholesterol and LDL-C levels also decreased to 146 mg/dL and 81 mg/dL, respectively. On the other hand, the serum anti-GM-CSF antibody remained persistently elevated despite the improvement in PAP (Table 1). No interventions other than statin therapy have been administered for PSP, and as of the current date, there has been no exacerbation of symptoms, with sustained improvement.

**Table 1:** Laboratory data before atorvastatin administration, 8 months after, and 16 months after administration.

	Before	8-month after	16-month after
LDH(U/L)	352	310	328
KL-6(U/mL)	12321	5195	3047
SP-D(ng/mL)	208	155	112
anti-GM-CSF antibody(U/mL)	93.7		141
AaDO_2_(Torr)	42.2		27

LDH, lactate dehydrogenase; SP-D, surfactant protein D.

## 3 Discussion

This report presents a case of successful treatment for untreated APAP using statins. While WLL is the standard treatment for APAP, it requires general anesthesia, and its complexity and safety due to the potential for severe hypopnea. In addition, international surveillance data indicates that WLL is performed an average of 2.5 ± 1.5 times on both right and left lungs, and in more than 10 % of cases, it is administered more than 5 times, highlighting the issue of recurrent treatment [2]. Consequently, WLL is primarily conducted in a limited number of specialized centers for severe APAP cases and often serves as the main symptomatic treatment for patients with mild or moderate disease.

Although PAP is often characterized by less pronounced symptoms compared to the notable imaging findings, approximately 50–90 % of patients experience dyspnea, which is the most common symptom [[3], [4], [5]]. It is also known that patients with APAP have an increased susceptibility to infections due to the accumulation of protein components in the alveolar space, resulting in reduced alveolar macrophage activity and an elevated risk of severe infections. A total of 58 % of APAP patients develop at least one infection, with a notably heightened risk of opportunistic infections, such as nocardiosis, being reported as well [6]. Even in the absence of severe disease, APAP significantly impacts patients' lives in terms of symptoms and complications, underscoring the necessity to identify simple, low-risk treatments applicable to mild and moderate cases of APAP.

There is a report describing two patients with refractory PAP complicated by hypercholesterolemia, in whom clinical symptoms, imaging findings, and respiratory function improved following statin treatment [7]. In a prospective observational study, 26 out of 40 (65 %) patients with PAP, who did not have hypercholesterolemia, responded to statins, of whom 4 patients had complete resolution of their imaging abnormalities and achieved normal respiratory function [8]. On the other hand, it should also be noted that approximately 30 % of APAP patients may undergo spontaneous resolution [9], which complicates the determination of whether the observed improvement is attributed to treatment or spontaneous resolution.

McCarthy et al. reported that GM-CSF plays a role in cholesterol clearance within alveolar macrophages and proposed that in APAP, anti-GM-CSF antibodies may reduce cholesterol clearance. Consequently, this interference could lead to decreased surfactant clearance within the alveoli, primarily due to accumulation of surfactants in the alveoli. Such accumulation may subsequently result in a secondary reduction in surfactant clearance, contributing to surfactant build-up within the alveoli [7]. Furthermore, their study also observed that statins exert a direct therapeutic impact on alveolar macrophages by enhancing the expression and efflux of cholesterol transporters within these cells. On the other hand, a single-center cohort study investigating cases of APAP that achieved remission through either WLL treatment or natural course reported a significant reduction in serum anti-GM-CSF antibody levels post-remission compared to pre-remission levels [10].

In the current case, following the oral administration of atorvastatin at a daily dose of 5 mg, there was a remarkable improvement in the patient's clinical symptoms, imaging findings, and lung function, resulting in a reduction of the DSS from 3 to 1. However, it is noteworthy that serum anti-GM-CSF antibody levels remained elevated and even appeared to increase upon reevaluation at the time of symptom improvement after atorvastatin treatment, in contrast to the above cohort study findings. Taken together, this suggests that the remission observed in this case may not be solely attributed to the natural course of the disease but could potentially be attributed to a direct effect of atorvastatin within the pulmonary alveoli.

Side effects of statin administration include myalgia, liver dysfunction and menstrual irregularities, but they are often minor and infrequent [11]. Compared with conventional treatments such as WLL, statins are simple and inexpensive.

## 4 Conclusion

In cases where hyperlipidemia coexists, statins may be considered a potential candidate for initial treatment in APAP.

## Key learning points

・ Statins are considered to reduce cholesterol accumulation in alveolar macrophages in APAP, thereby improving surfactant clearance by alveolar macrophages and exerting therapeutic effects.
・ Following the oral administration of atorvastatin, there was a remarkable improvement in the patient's clinical symptoms, imaging findings, and lung function.
・ Statin administration may be considered as one of the treatment options for APAP.

## CRediT authorship contribution statement

**So Shimamura:** Writing – original draft. **Honami Morikawa:** Writing – review & editing. **Ken Shinohara:** Writing – review & editing. **Hiroki Ohkoshi:** Writing – review & editing. **Chisa Omori:** Writing – review & editing. **Yuuki Hoshino:** Writing – review & editing. **Yoshinori Uchida:** Writing – review & editing. **Saiki Masafumi:** Writing – review & editing. **Shinnosuke Ikemura:** Writing – review & editing. **Naoki Ohishi:** Writing – review & editing. **Tetsuo Kondo:** Writing – review & editing. **Kenzo Soejima:** Writing – review & editing.

## Declaration of competing interest

The authors declare that they have no known competing financial interests or personal relationships that could have appeared to influence the work reported in this paper.
